# Supplementary material for: PAK4 suppresses RELB to prevent senescence-like growth arrest in breast cancer
Source: Nat Commun. 2019 Aug 9;10:3589. doi: 10.1038/s41467-019-11510-4 (PMC6689091; doi:10.1038/s41467-019-11510-4)
Supplement: Supplementary file 1 — Supplementary Information [file 41467_2019_11510_MOESM1_ESM.pdf]

Supplementary Information

PAK4 suppresses RELB to prevent senescence-like growth arrest in breast cancer

Costa *et al.*

Supplementary Figures 1 - 7

Supplementary Tables 1 - 5

Supplementary References

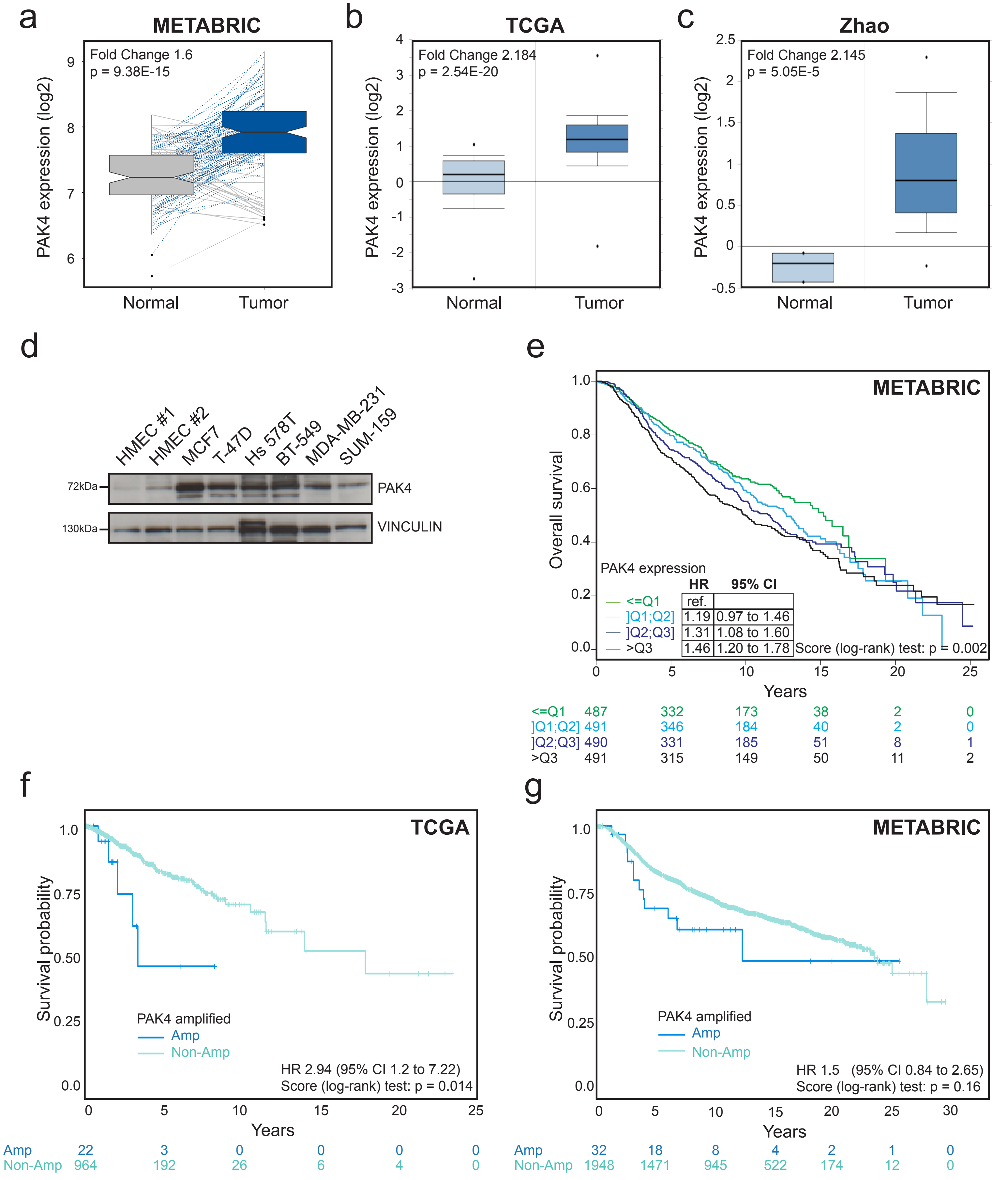

Supplementary Figure 1

Supplementary Figure 1

PAK4 overexpression in breast cancer is associated with unfavorable outcome.

(a) PAK4 mRNA expression in paired breast carcinomas and normal breast tissues (n = 131 patients / 263 samples) in the METABRIC dataset.

(b) PAK4 expression in breast carcinomas (n = 393) and in normal breast tissues (n = 61) in the TCGA breast cancer cohort.

(c) PAK4 expression in breast carcinomas (n = 38) and in normal breast tissues (n = 3) in the Zhao breast cancer cohort.

(d) Representative immunoblot of whole cell lysates derived from the indicated human breast cancer cell lines and two independent batches of their normal counter parts human mammary epithelial cells (HMEC #1 and #2). The membrane was probed with antibodies against PAK4 using VINCULIN as loading control.

(e) Kaplan–Meier plot and analysis of overall survival in the METABRIC cohort stratified according to quartiles of PAK4 expression.

(f) Kaplan–Meier plot and survival probability in the TCGA cohort stratified according to *PAK4* gene amplification status.

(g) Kaplan–Meier plot and survival probability in the METABRIC cohort stratified according to *PAK4* gene amplification status.

In (**a**), (**b**) and (**c**) data are represented as boxplots where the middle line is the median, the lower and upper hinges correspond to the first and third quartiles, the upper whisker extends from the hinge to the largest value no further than 1.5 x IQR from the hinge (where IQR is the inter-quartile range) and the lower whisker extends from the hinge to the smallest value at most 1.5 x IQR of the hinge, while data beyond the end of the whiskers are outlying points that are plotted individually. The Fold Change and the p-value of a paired Wilcoxon test are indicated in **(a)**. The Fold Change and the p-values of Mann-Whitney tests are indicated in **(b)** and **(c)**. The Hazard Ratio (HR), 95 % confidence intervals (CI) and p-values by the log-rank (Mantel-Cox) test are indicated in **(e)**, **(f)** and **(g)**. n is specified for each patient subgroup below (**e**), (**f**) and (**g**).

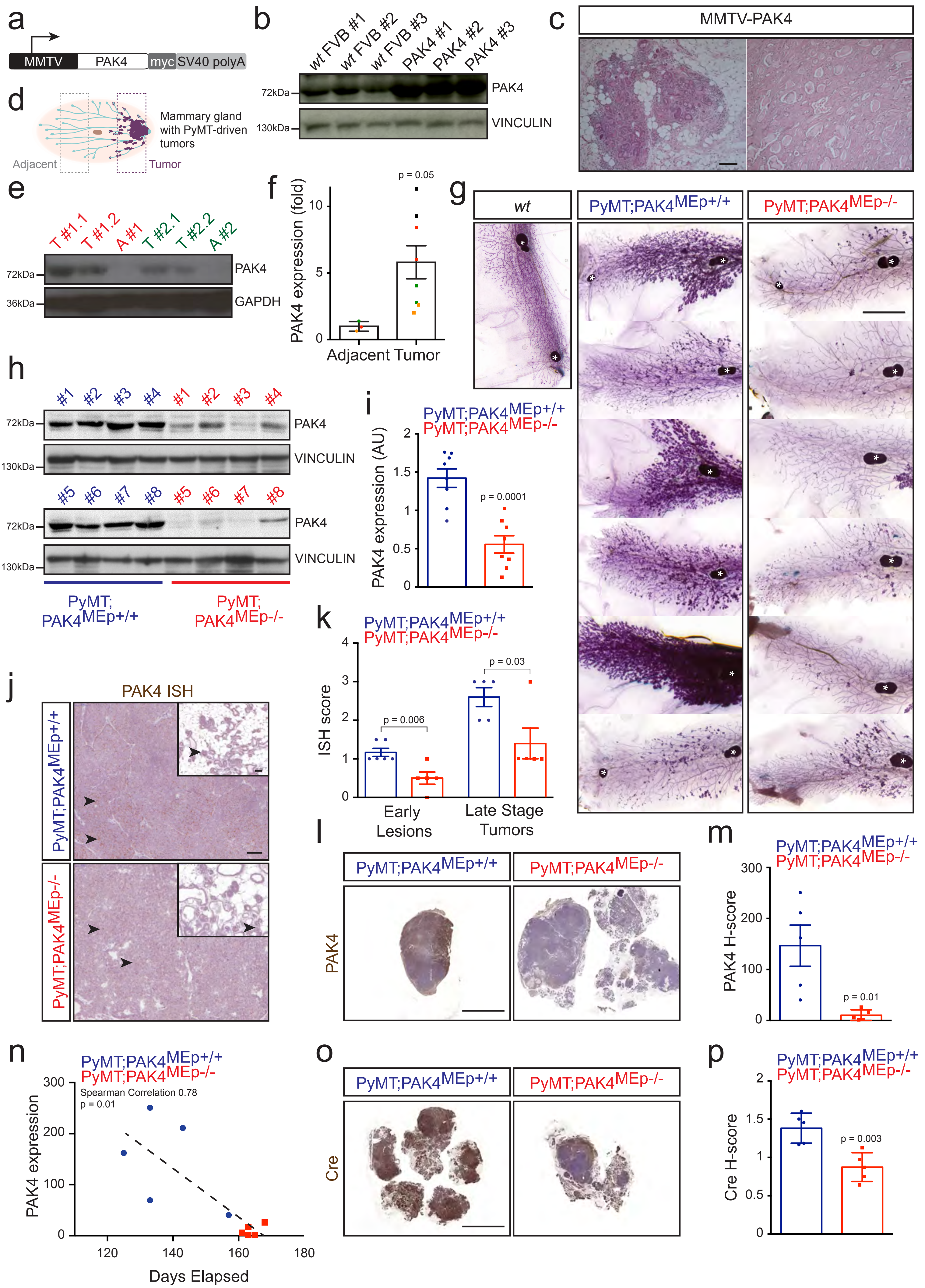

Supplementary Figure 2

Supplementary Figure 2

PAK4 promotes murine mammary tumorigenesis.

(a) Illustration of the linearized plasmid used to generate MMTV-PAK4.

(b) Representative immunoblot of PAK4 expression in mammary glands from 12 weeks old mice of the indicated genotypes (n = 3).

(c) Representative morphologies of H&E-stained MMTV-PAK4 tumors. Scale bar, 250 μm.

(d) Schematic illustration depicting the sampling of PyMT-driven mammary tumors and paired tumor-adjacent mammary tissue.

(e-f) Representative immunoblot (**e**) and quantification (**f**) of PAK4 abundance in PyMT-driven mammary tumor extracts (T, n = 8) and paired tumor-adjacent mammary tissue (A, n = 3). Colored dots depict matched tissue pairs.

(g) Carmine-stained wholemounts of inguinal mammary glands from 12 weeks old mice (n = 6 per genotype) quantified in **Figure 1c**. Scale bar, 5 mm. An age matched *wt* gland is shown for comparison. White asterisks denote lymph nodes.

(h-i) Immunoblots (**h**) and quantification (**i**) of PAK4 abundance in PyMT;PAK4$^{MEp+/+}$ and PyMT;PAK4$^{MEp-/-}$ mammary tumor extracts (n = 8 per genotype).

(j) Representative *in situ* hybridization of *PAK4* mRNA (arrowheads) in late stage tumors of the indicated genotypes. Insets show adjacent mammary tissue. Scale bar, 100 μm.

(k) Quantification of *PAK4* mRNA expression in lesions (8 to 11 weeks old) and tumors from PyMT;PAK4$^{MEp+/+}$ (lesions n = 6; tumors n = 5) and PyMT;PAK4$^{MEp-/-}$ (lesions n = 5; tumors n = 5).

(l-m) Representative PAK4 immunostaining (**l**) and quantification (**m**) in the solid area of tumors (n = 5 per genotype). Scale bar, 5mm.

(**n**) Correlation between PAK4 expression determined by immunohistochemistry and overall survival.

(o-p) Representative Cre immunostaining (**o**) and quantification (**p**) in whole tissue sections of tumors (n = 5 per genotype). Scale bar, 5mm.

Data are represented as mean ± SEM in **(f)**, **(i)**, **(k)**, **(m)** and **(p)** and the p-values by two-tailed unpaired *t* test are indicated. The p-value has been corrected for multiple comparisons using the Holm-Sidak method in (**k**). The Spearman correlation and p-value by Spearman's test are indicated in (**n**). Membranes were probed with the indicated antibodies in **(b)**, **(e)** and **(h)** using GAPDH or VINCULIN as a loading control.

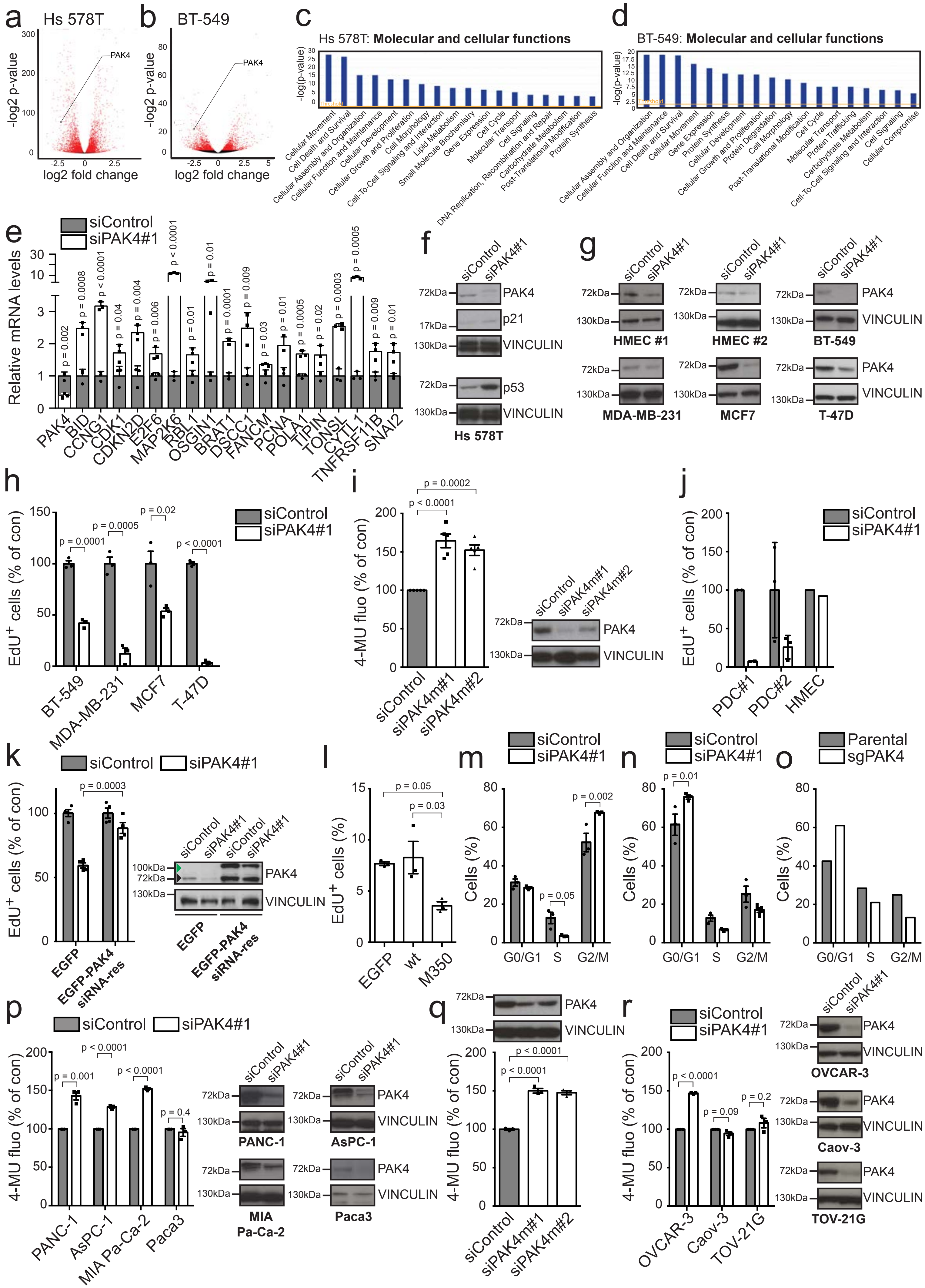

Supplementary Figure 3

Supplementary Figure 3

PAK4 depletion induces senescence-like growth arrest in cancer cells.

(a-b) Volcano plots of differentially expressed genes (red) by RNA-Seq of indicated cells 3 days after siPAK4 transfection.

(c-d) Top altered molecular and cellular functions by Ingenuity pathway analyses of the RNA-Seq datasets.

(e) RT-qPCR of Hs 578T mRNA 72 hours after transfection (n = 3) for validation of hits involved in cell cycle arrest, DNA-damage/repair and SASP.

(f-g) Representative immunoblots of denoted cells 5 days after transfection. HMECs were exposed longer than cancer cells due to lower PAK4 expression.

(h) Quantification of EdU^+ cells 5 days after transfection (n = 3).

(i) Quantification of SA-β-gal activity in PyMT-derived cells 5 days after transfection (n = 5) and representative immunoblot.

(j) Quantification of EdU^+ cells in primary patient-derived breast cancer cells (PDC#1 n = 1 and PDC#2 n = 2) and HMECs (n = 1) 5 days after transfection.

(k) Quantification of EdU^+ cells, 4 days after transfection of MDA-MB-231 stably expressing EGFP or EGFP-PAK4-siRNA-resistant mutant (n = 4) and representative immunoblot of endogenous (black arrow) and EGFP-tagged (green arrow) PAK4 forms.

(l) Quantification of EdU^+ cells among high expressing EGFP, EGFP-PAK4 *wt*, or EGFP-PAK4-M350 MCF7 cells, 2 days after transfection (n = 3).

(m-o) Cell cycle profiles of Hs 578T (**m**) and MCF-7 (**n**) cells 5 days after transfection (n = 3) and BT-549 cells (**o**) 10 days after transduction (n = 1).

(p-r) Quantification of SA-β-gal activity in human pancreatic (**p**), mouse KPC-derived (**q**) and in human ovarian (**r**), cancer cells and representative immunoblots 5 days after transfection (n = 3).

Data are represented as mean ± SEM in (**e**), (**h-i**), (**k-n**) and (**p-r**) and as mean ± SD in (**j**). p-values by two-tailed unpaired *t* tests with correction for multiple comparisons using the Holm-Sidak method are indicated in (**e**), (**h**), (**p**) and (**r**); 1-way ANOVA followed by Dunnett's *post hoc* test in (**i**) and (**q**); 2-way ANOVA followed by Tukey's *post hoc* test in (**k**); by 1-way ANOVA followed by Tukey's *post hoc* test in (**l**) and by 2-way ANOVA followed by Sidak's multiple comparison test in (**m**) and (**n**).

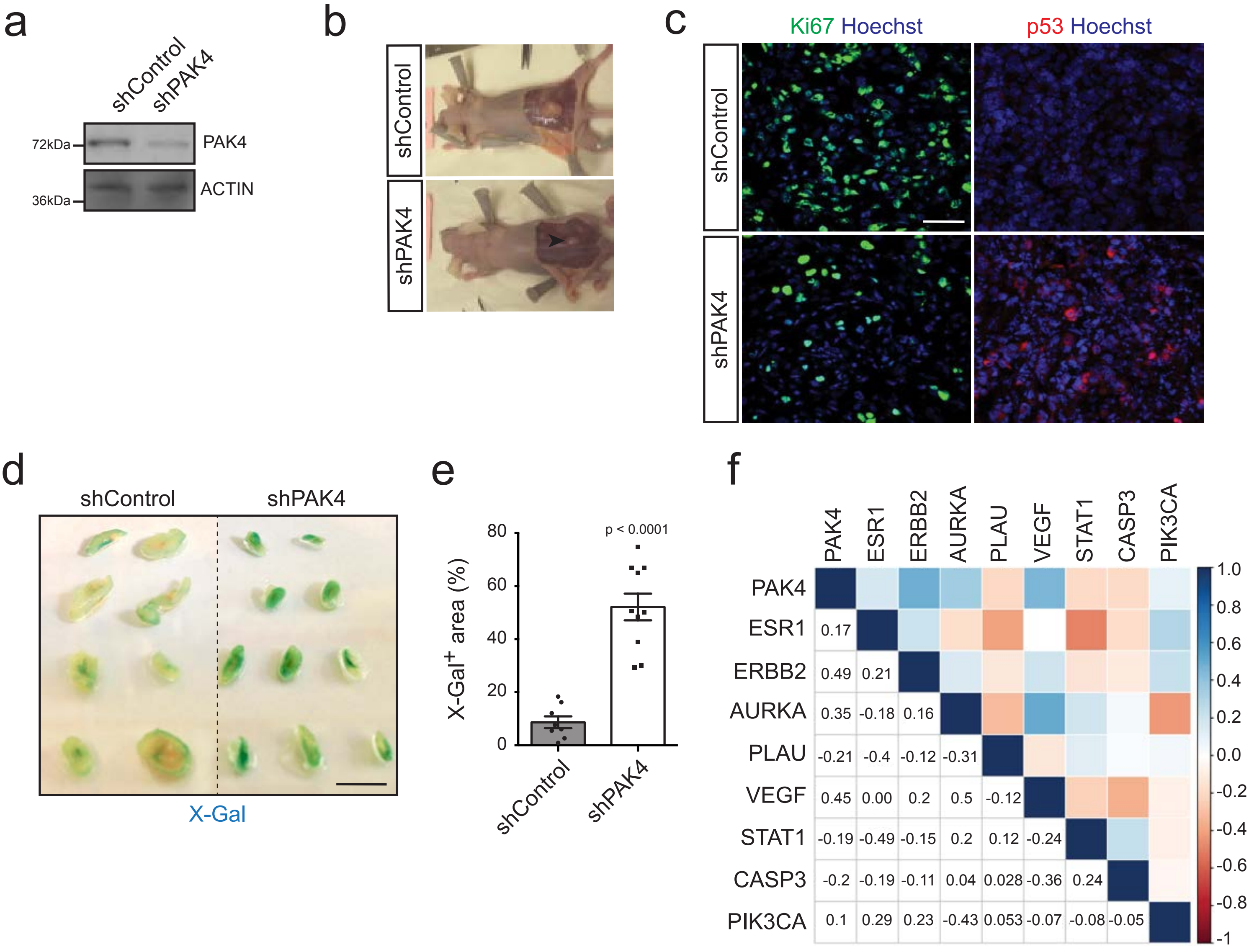

Supplementary Figure 4

Supplementary Figure 4

PAK4 inhibition induces senescence-like programs *in vivo*.

(a) Representative PAK4 immunoblot (with ACTIN as loading control) of MCF7 cells stably expressing control shRNA or PAK-shRNA prior to subcutaneous injection into nude mice.

(b) Representative images of tumor-bearing mice (arrowheads) of the indicated groups at the experimental endpoint.

(c) Representative images of Ki67- and p53-stained tumor sections of the indicated groups. Scale bar, 50 μm.

(d) X-Gal-stained tumor slices derived from xenografts of MCF7 cells stably expressing control shRNA or PAK-shRNA.

(e) Quantification of the X-Gal^{+} area in tumor slices of MCF7 xenografts (shControl n = 8 and shPAK4 n = 10).

(f) Spearman correlation in the METABRIC dataset between expression level of PAK4 and known expression modules reflecting biological processes in breast cancer. The Spearman correlation values are indicated (n = 1992, ESR1: Estrogen Receptor Signaling; ERBB2: HER2 Signaling; AURKA: Proliferation; PLAU: Invasion; VEGF: Angiogenesis; STAT1: Immune response; CASP3: Apoptosis and PIK3CA: PIK3CA Signaling).

Data are represented as mean ± SEM in (**e**) and the p-value by two-tailed unpaired *t* test is indicated.

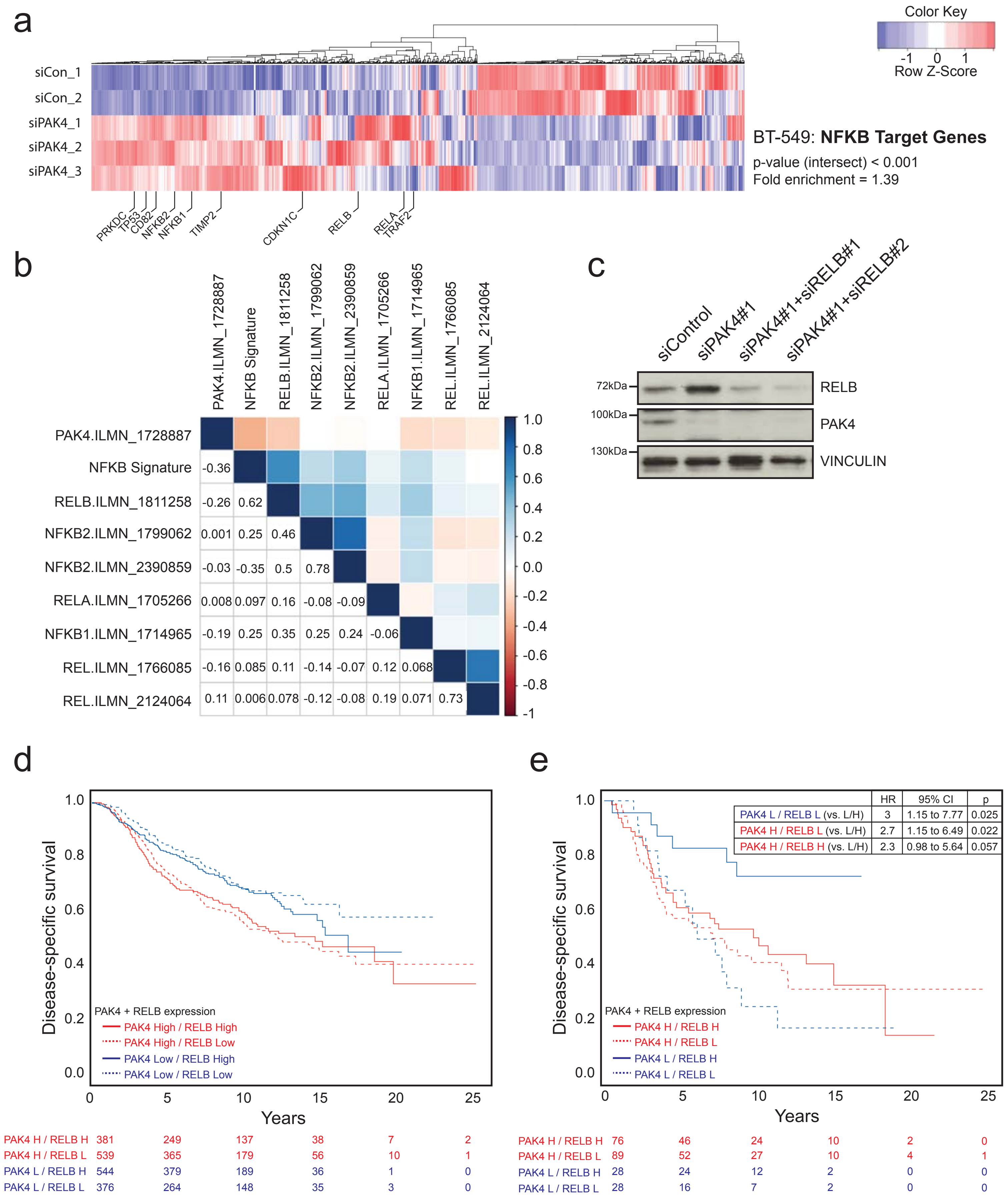

Supplementary Figure 5

Supplementary Figure 5

PAK4 suppresses senescence-like growth arrest via the NF-κB subunit RELB.

(a) Column clustered heatmaps of NF-κB (RELA) target genes that are differentially expressed in BT-549 cells upon siPAK4. Genes are by column and samples by row. The color intensity represents column Z-score, where red indicates highly and blue lowly expressed. Selected NF-κB target genes are indicated.

(b) Correlation between expression level of PAK4 and NF-κB subunits in the METABRIC dataset (n = 1992). An NF-κB signature was also added to the correlation matrix. The Spearman correlation values are indicated.

(c) Representative immunoblots of PAK4 and RELB knockdown efficiency in Hs 578T cells 4 days after transient transfection with the indicated siRNAs using VINCULIN as a loading control.

(d) Kaplan–Meier plot and analysis of disease-specific survival of breast cancer patients of the METABRIC cohort stratified according to median PAK4/RELB expression.

(e) Kaplan-Meier plot and analysis of disease-specific survival of breast cancer patients of the HER2-enriched subtype of the METABRIC cohort stratified based on PAK4 and RELB expression levels.

n is specified for each patient subgroup below (**d**) and (**e**) and the median PAK4 and RELB expression were defined as high or low (H, i.e. above the median; L, i.e. below the median). The Hazard Ratio (HR), 95 % confidence intervals (CI) and p-values are indicated in (**e**) by the log-rank (Mantel-Cox) test.

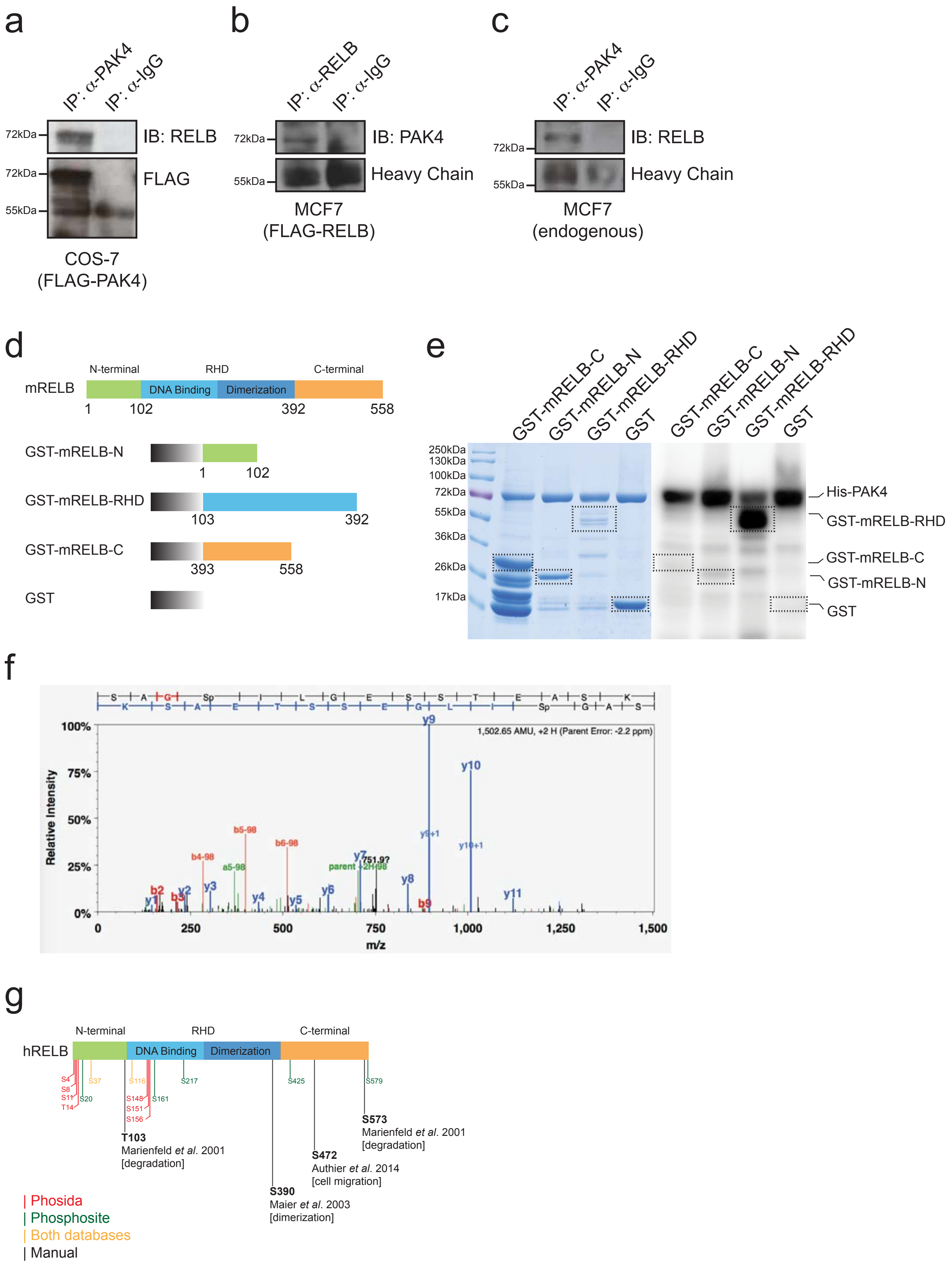

Supplementary Figure 6

Supplementary Figure 6

PAK4-mediated RELB phosphorylation on Ser151

(a) Immunoblot (IB) analysis as indicated of overexpressed PAK4-immunoprecipitates (IP) derived from COS-7 cells, using IgG-IP as control.

(b) Immunoblot analysis as indicated of overexpressed RELB-immunoprecipitates derived from MCF7 cells, using IgG-IP as control.

(c) Immunoblot analysis as indicated of endogenous PAK4-immunoprecipitates derived from MCF7 cells, using IgG-IP as control.

(d) Schematic illustration of the purified GST-tagged murine RELB fragments N-terminus (GST-mRELB-N), C-terminus (GST-mRELB-C) and REL-homology domain (GST-mRELB-RHD).

(e) Radioactive kinase assay with recombinant GST-tagged murine RELB fragments. Coomassie-stained gel is shown to the left as loading control.

(f) Mass spectrometry peaks identifying Ser151 as a putative PAK4 phosphorylation site. GST-tagged human RELB aa 102-400 (GST-RHD) was phosphorylated by His-PAK4 *in vitro* and analyzed by mass spectrometry.

(g) Schematic annotation of detected and validated phosphorylation sites within RELB. The human gene was used for search in Phosida and PhosphoSitePlus databases and the literature curated for additional residues. The scheme is annotated for indicated functions. Note that residues previously identified using mouse RELB were converted to the corresponding human residue for display.

Results in (**a**), (**b**), (**c**) and (**e**) are representative of three independent repeats.

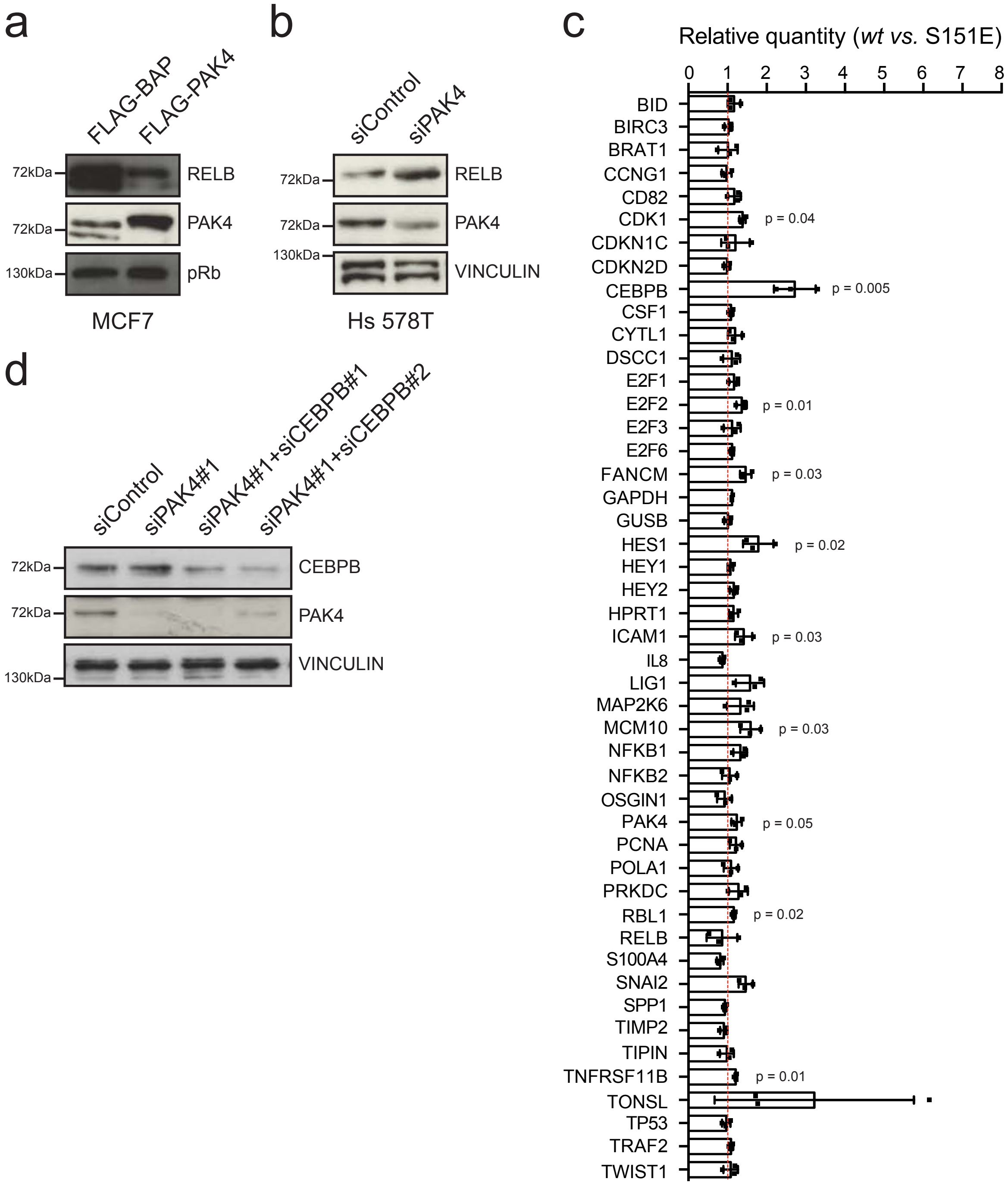

Supplementary Figure 7

Supplementary Figure 7

RELB-Ser151 controls DNA binding and transcription

(a) Representative immunoblots of nuclear lysates used in the ELISA-based measurements of RELB-DNA binding in MCF7 cells stably expressing the indicated FLAG-tagged constructs.

(b) Representative immunoblots of nuclear lysates used in the ELISA-based measurements of RELB-DNA binding, 4 days after transfection of the indicated siRNAs in Hs 578T cells.

(c) RT-qPCR array of Hs 578T cells transfected with FLAG-tagged RELB-S151E and RELB-*wt*. mRNA was prepared 24 hours after transient transfection (n = 3 per group), followed by quantification of mRNA expression of the indicated genes.

(d) Representative immunoblots of knockdown efficiency of CEBP/β and PAK4 in Hs 578T cells, 4 days after transient transfection with the indicated siRNAs.

Data in **(c)** are represented as mean ± SEM of RELB-*wt* relative to RELB-S151E and the p-values by two-tailed unpaired *t* tests with correction for multiple comparisons using the Holm-Sidak method are indicated. Membranes in (**a**), (**b**) and (**d**) were probed with the indicated antibodies using pRB or VINCULIN as loading controls.

Supplementary Table 1 | General information about the breast cancer cell lines used in the study

Cell line	Origin	ER	PR	HER2	PAM50	Metastatic[4]	COSMIC database of cell line mutations, restricted to Cancer gene Census	CNA								
								PAK4	HRAS	KRAS	NRAS	ARAF	BRAF	RAF1	TP53	RB1
**BT-549**	Primary tumor	-	-	-	Basal-like	Yes	BUB1B, CYLD, DCTN1, FGFR1, MUTYH, PCM1, POLQ, PTEN, PTPRT, RNF213, SETBP1, **TP53 (R249S***)**, USP8	-	-	-	-	-	-	-	-	-
**Hs 578T**	Primary tumor	-	-	-	Basal-like	Yes	CBFA2T3, EPAS1, **HRAS (G12D*)**, NF1, PIK3R1, POLE, POLQ, RANBP2, **TP53 (V157F****)**	-	-	-	-	-	-	-	-	-
**MCF7**	Pleural effusion	+	+	-	Luminal A	No	ATP2B3, BCR, CLIP1, DNM2, EP300, ERBB4, FLT3, GATA3, MAP3K13, MYH9, NBN, PALB2, PAX5, PIK3CA	-	Deep del	-	Amp	-	-	-	-	-
**MDA-MB-231**	Pleural effusion	-	-	-	Basal-like	Yes	AR, ATM, AXIN2, **BRAF (G464V**)**, CASP8, CD79A, CTCL1, CRTC3, DROSHA, EXT2, GAS7, GNAS, HOXD3, IRS4, **KRAS (G13D*)**, NF1, NF2, PBRM1, PDGFRA, PER1, SLC45A3, TCEA1, **TP53 (R280K*****)**	-	-	-	-	-	-	-	-	-
**T-47D**	Pleural effusion	+	+	-	Luminal B	No	ACVR1, ARID1A, DDR2, MLLT4, TCP1, PDE4DIP, PIK3CA, SPEN, STAT3, **TP53 (L194F***)**	-	-	-	-	-	-	-	-	-

Deep del = Deep deletion, Amp = Amplification, ER = Estrogen receptor, PR = Progesterone receptor, HER2 = ERBB2 receptor, CNA = copy number alteration
*Gain-of-function (OncoKB), recurrent hotspot (Cancer Hotspots), 3D clustered hotspot (3D Hotspots); **Gain-of-function (OncoKB), 3D clustered hotspot (3D Hotspots); ***Likely loss-of-function (OncoKB), recurrent hotspot (Cancer Hotspots), 3D clustered hotspot (3D Hotspots); ****Likely loss-of-function (OncoKB), recurrent hotspot (Cancer Hotspots); *****Loss-of-function (OncoKB), recurrent hotspot (Cancer Hotspots), 3D clustered hotspot (3D Hotspots)

Supplementary Table 2 | Multivariate Cox proportional hazards analyses for disease-specific survival in association with dichotomized PAK4 expression in the METABRIC cohort

	HR	95 % CI	p-value
**PAK4 High (vs. Low)**	1.30	0.64 to 0.94	0.008
**Tumor size > 20mm (vs. ≤ 20)**	1.61	1.32 to 1.96	<0.001
**Lymph node N+ (vs. N0)**	2.28	1.87 to 2.78	<0.001
**Tumor grade 2 (vs. grade 1)**	1.40	0.86 to 2.30	0.18
**Tumor grade 3 (vs. grade 1)**	1.50	0.91 to 2.48	0.11
**Age > 45y and ≤ 55y (vs. ≤ 45y)**	0.84	0.62 to 1.14	0.26
**Age > 55y (vs. ≤ 45y)**	0.97	0.75 to 1.27	0.85
**PAM50 Basal-like (vs. Luminal A)**	2.15	1.57 to 2.95	<0.001
**PAM50 Her2-like (vs. Luminal A)**	2.28	1.68 to 3.10	<0.001
**PAM50 Luminal B (vs. Luminal A)**	2.00	1.54 to 2.61	<0.001
**PAM50 Normal-like (vs. Luminal A)**	1.91	1.31 to 2.77	<0.001

CI = confidence interval, HR = hazard ratio.

Supplementary Table 3 | Multivariate Cox proportional hazards analyses for overall survival in association with dichotomized PAK4 expression in the METABRIC cohort

	HR	95 % CI	p-value
**PAK4 High (vs. Low)**	1.16	0.75 to 1.00	0.043
**Tumor size > 20mm (vs. ≤ 20)**	1.47	1.27 to 1.71	<0.001
**Lymph node N+ (vs. N0)**	1.75	1.51 to 2.02	<0.001
**Tumor grade 2 (vs. grade 1)**	1.11	0.82 to 1.50	0.49
**Tumor grade 3 (vs. grade 1)**	1.22	0.90 to 1.67	0.20
**Age > 45y and ≤ 55y (vs. ≤ 45y)**	0.81	0.61 to 1.07	0.13
**Age > 55y (vs. ≤ 45y)**	1.42	1.13 to 1.79	0.0024
**PAM50 Basal-like (vs. Luminal A)**	1.47	1.16 to 1.86	0.0013
**PAM50 Her2-like (vs. Luminal A)**	1.46	1.16 to 1.83	0.0011
**PAM50 Luminal B (vs. Luminal A)**	1.38	1.15 to 1.67	<0.001
**PAM50 Normal-like (vs. Luminal A)**	1.32	1.01 to 1.74	0.044

CI = confidence interval, HR = hazard ratio.

Supplementary Table 4 | *PAK4* amplification status in breast cancer datasets

	PAK4 Amplified (n)	Total (n)	Percentage (%)
**TCGA**	22	987	2.23
**METABRIC**	32	1980	1.62

Supplementary Table 5 | List of primers

Primer	Sequence
MMTV-PAK4 Forward	TTTGGAAAGAAGAAGAAGC
MMTV-PAK4 Reverse	ACTGCCACTGCCTGCCTCAC
Cre Forward	GGTTTCCCGCAGAACCTGAAG
Cre Reverse	GCTAGTGCCTTCTCTACACC
PyMT Forward	GGAAGCAAGTACTTCACAAGGG
PyMT Reverse	GGA AAG TCA CTA GGA GCA
PAK4 Forward	CGGATATTGTCACCCACACCA
PAK4 Reverse	CTAACAGGGACAGGAGCT
FLAG-RELB Forward	TAGAAGCTTATGCTTCGGTCTGGGCCAGCC
FLAG-RELB Reverse	GTAGGTACCCTACGTGGCTTCAGGCCCCGGGG
S151A Forward	GAGGGCCGCTCGGCCGGCGCCATCCTTGGGGAGAGC
S151A Reverse	GCTCTCCCCAAGGATGGCGCCGGCCGAGCGGCCCTC
S151E Forward	GAGGGCCGCTCGGCCGGCGAGATCCTTGGGGAGAGC
S151E Reverse	GCTCTCCCCAAGGATCTCGCCGGCCGAGCGGCCCTC
GST-RELB Forward	AAGGTAGAATTCGCCACGCCGCCGCCTTGG
GST-RELB Reverse	GAGTAGCGGCCGCCTAGCGAGGCAGGTACGTGAAAGG
PAK4 siRNA-resistant Forward	GCTCTTCAACGAGGTTGTTATCATGAGGGAC
PAK4 siRNA-resistant Reverse	GTAGTCCCTCATGATAACAACCTCGTTGAAG

Supplementary References

1 Marienfeld, R. *et al.* Signal-specific and phosphorylation-dependent RelB degradation: a potential mechanism of NF-kappaB control. *Oncogene* **20**, 8142-8147, doi:10.1038/sj.onc.1204884 (2001).

2 Maier, H. J., Marienfeld, R., Wirth, T. & Baumann, B. Critical role of RelB serine 368 for dimerization and p100 stabilization. *The Journal of biological chemistry* **278**, 39242-39250, doi:10.1074/jbc.M301521200 (2003).

3 Authier, H. *et al.* IKK phosphorylates RelB to modulate its promoter specificity and promote fibroblast migration downstream of TNF receptors. *Proceedings of the National Academy of Sciences of the United States of America* **111**, 14794-14799, doi:10.1073/pnas.1410124111 (2014).

4 Smith, S. E. *et al.* Molecular characterization of breast cancer cell lines through multiple omic approaches. *Breast cancer research : BCR* **19**, 65, doi:10.1186/s13058-017-0855-0 (2017).
