# Supplementary material for: PAK4 suppresses RELB to prevent senescence-like growth arrest in breast cancer
Source: Nat Commun. 2019 Aug 9;10:3589. doi: 10.1038/s41467-019-11510-4 (PMC6689091; doi:10.1038/s41467-019-11510-4)
Supplement: Supplementary file 3 — Description of Additional Supplementary Files [file 41467_2019_11510_MOESM3_ESM.pdf]

Description of Additional Supplementary Files

File Name: Supplementary Data 1
Description: Exome sequencing of MMTV-PAK4 tumors.

File Name: Supplementary Data 2
Description: Alignment statistics of RNA-Seq data in Hs 578T and BT-549 cells.

File Name: Supplementary Data 3
Description: Differential expression analysis of RNA-seq data between siControl and siPAK4#1 in Hs 578T cells, 72 hours after transient transfection.

File Name: Supplementary Data 4
Description: Differential expression analysis of RNA-seq data between siControl and siPAK4#1 in BT-549 cells, 72 hours after transient transfection.

File Name: Supplementary Data 5
Description: NF-κB (RELA) target genes in Hs 578T cells upon PAK4 knockdown.

File Name: Supplementary Data 6
Description: NF-κB (RELA) target genes in BT-549 cells upon PAK4 knockdown.
