# Supplementary material for: PAK4 suppresses RELB to prevent senescence-like growth arrest in breast cancer
Source: Nat Commun. 2019 Aug 9;10:3589. doi: 10.1038/s41467-019-11510-4 (PMC6689091; doi:10.1038/s41467-019-11510-4)
Supplement: Supplementary file 10 — Reporting Summary [file 41467_2019_11510_MOESM10_ESM.pdf]

nature research

Corresponding author(s): Staffan Strömblad
Last updated by author(s): Jun 4, 2019

Reporting Summary

Nature Research wishes to improve the reproducibility of the work that we publish. This form provides structure for consistency and transparency in reporting. For further information on Nature Research policies, see Authors & Referees and the Editorial Policy Checklist.

Statistics

For all statistical analyses, confirm that the following items are present in the figure legend, table legend, main text, or Methods section.

n/a	Confirmed	
☐	☒	The exact sample size (*n*) for each experimental group/condition, given as a discrete number and unit of measurement
☐	☒	A statement on whether measurements were taken from distinct samples or whether the same sample was measured repeatedly
☐	☒	The statistical test(s) used AND whether they are one- or two-sided<br>*Only common tests should be described solely by name; describe more complex techniques in the Methods section.*
☐	☒	A description of all covariates tested
☐	☒	A description of any assumptions or corrections, such as tests of normality and adjustment for multiple comparisons
☐	☒	A full description of the statistical parameters including central tendency (e.g. means) or other basic estimates (e.g. regression coefficient) AND variation (e.g. standard deviation) or associated estimates of uncertainty (e.g. confidence intervals)
☐	☒	For null hypothesis testing, the test statistic (e.g. *F*, *t*, *r*) with confidence intervals, effect sizes, degrees of freedom and *P* value noted<br>*Give P values as exact values whenever suitable.*
☒	☐	For Bayesian analysis, information on the choice of priors and Markov chain Monte Carlo settings
☒	☐	For hierarchical and complex designs, identification of the appropriate level for tests and full reporting of outcomes
☐	☒	Estimates of effect sizes (e.g. Cohen's *d*, Pearson's *r*), indicating how they were calculated

Our web collection on statistics for biologists contains articles on many of the points above.

Software and code

Policy information about availability of computer code

Data collection	NIS-Elements software (Nikon) was used for image acquisition.
Data analysis	ImageJ/Fiji and Quantity One (Bio-Rad) were used for western blot band quantification.<br>ImageJ/Fiji and NIS-Elements (Nikon) softwares were used for image analyses.<br>GraphPad Prism 6.0 and R statistical software v3.1.0 were used for for statistical analyses.<br>Tophat2, DESeq2, Cufflinks, the ggplot2 package in R and Ingenuity Pathway Analysis (IPA, Qiagen) were used for RNA-seq data analyses.

For manuscripts utilizing custom algorithms or software that are central to the research but not yet described in published literature, software must be made available to editors/reviewers. We strongly encourage code deposition in a community repository (e.g. GitHub). See the Nature Research guidelines for submitting code & software for further information.

Data

Policy information about availability of data

All manuscripts must include a data availability statement. This statement should provide the following information, where applicable:
- Accession codes, unique identifiers, or web links for publicly available datasets
- A list of figures that have associated raw data
- A description of any restrictions on data availability

The RNA-Seq data reported here has been deposited in the NCBI Geo under ID code: GSE112817. Exome-Seq data are available in SRA under the accession code PRJNA545882. Remaining primary data of interest is provided in the source data file of this paper.

October 2018

Field-specific reporting

Please select the one below that is the best fit for your research. If you are not sure, read the appropriate sections before making your selection.

☒ Life sciences ☐ Behavioural & social sciences ☐ Ecological, evolutionary & environmental sciences

For a reference copy of the document with all sections, see nature.com/documents/nr-reporting-summary-flat.pdf

Life sciences study design

All studies must disclose on these points even when the disclosure is negative.

Sample size	Group size for original data created here was based on previous experience. No statistical method was used to predetermine sample size.
Data exclusions	The replicate BT-549 siControl #3 was incorrectly sequenced and it was therefore removed from the RNA-Seq analyses.
Replication	Unless explicitly stated, all data shown were obtained from at least 3 biological independent experiments. For representative images, each experiment was successfully repeated at least three times under similar conditions.
Randomization	Animals were randomly assigned to experimental groups.
Blinding	All mouse xenograft experiments were done in a blinded fashion.

Reporting for specific materials, systems and methods

We require information from authors about some types of materials, experimental systems and methods used in many studies. Here, indicate whether each material, system or method listed is relevant to your study. If you are not sure if a list item applies to your research, read the appropriate section before selecting a response.

Materials & experimental systems

n/a	Involved in the study	
☐	☒	Antibodies
☐	☒	Eukaryotic cell lines
☒	☐	Palaeontology
☐	☒	Animals and other organisms
☒	☐	Human research participants
☒	☐	Clinical data

Methods

n/a	Involved in the study	
☒	☐	ChIP-seq
☒	☐	Flow cytometry
☒	☐	MRI-based neuroimaging

Antibodies

Antibodies used	The following antibodies were used for immunostaining: AF488-conjugated α-p53 (1:100, DO-1 #sc-126 AF488, Santa Cruz), α-Ki67 (1:250, #ab15580, AbCam), α-BrdU mAb G3G4 (G3G4, Developmental Studies Hybridoma Bank, Department of Biological Sciences, University of Iowa, Iowa City, IA), α-Cre antibody (1:100, PRB-106P, Covance) and newly generated α-PAK4 #73 antibody diluted 1:100.<br>Western Blot membranes were probed with the following primary antibodies: α-PAK4 pab 6508 (1:1000), α-RELB (1:1000, C1E4 #4922, Cell Signaling; 1:1000, #06-1105, Millipore; 1:1000, EP613Y #GTX61291, Genetex and 1:1000, clone 17.3 #LS-C354950-100, LSBio), α-p53 (1:1000, DO-1 #sc-126, Santa Cruz); α-p21 (1:1000, C19 #sc-397, Santa Cruz); α-ACTIN (1:1000, JLA20, Developmental Studies Hybridoma Bank - DSHB); α-VINCULIN (1:100000, #V9131, Sigma); α-GAPDH (1:50000, #MAB374, Millipore) and α-pRb (1:1000, #554136, BD-Pharmingen).<br>FLAG-tagged proteins were immunoprecipitated with EZview Red ANTI-FLAG M2 Affinity Gel (F2426, Sigma). Otherwise, the following antibodies were used: monoclonal mouse α-PAK4 (clone OTI1C7 #CF807297, Origene), monoclonal mouse α-RELB (clone 17.3 #LS-C354950-100, LSBio) and α-mouse IgG (I5381, Sigma) as control.
Validation	Validation statement for each primary antibody is provided on the manufacturer's website. Validation of anti-PAK4 antibodies here generated was performed through immunoblotting of lysates from cells with or without transfection with siPAK4.

Eukaryotic cell lines

Policy information about cell lines

Cell line source(s)	HMEC (batches #1 and #2), Hs 578T, BT-549, MCF7, MDA-MB-231, T-47D, OVCAR-3, Caov-3 and TOV-21G were obtained from the American Type Culture Collection (ATCC). SUM-159 were obtained from Asterand. AsPC-1, PANC-1 and MIA Pa-Ca-2 were obtained from the European Collection of Authenticated Cell Cultures (ECACC). These pancreatic cancer cells and Paca3 (Moore et al., 2001) were a generous gift from Rainer Heuchel and Matthias Löhr.

Authentication	Cells were not subjected to additional cell authentication.
Mycoplasma contamination	Cells were tested for mycoplasma. All cell lines were found negative with the exception of stably transfected MCF7 cells.
Commonly misidentified lines (See ICLAC register)	N/A

Animals and other organisms

Policy information about studies involving animals; ARRIVE guidelines recommended for reporting animal research

Laboratory animals	MMTV-PAK4 were generated in inbred FVB/N strain.<br>PAK4fl/fl mice (B6.129S2(FVB)-Pak4tm2.1Amin/J, gift from Audrey Minden) (Tian et al., 2009) were crossed with MMTV-Cre/Line D mice (Tg(MMTV-cre)4Mam/J, Jackson Laboratory) (Wagner et al., 1997) and then bred to MMTV-PyMT (B6.FVB-Tg(MMTV-PyVT)634Mul/LellJ, gift from Lars Holmgren) (Davie et al., 2007). All mice were maintained on B6 background.<br>Female 10 to 15 weeks old MMTV-PyMT (FVB/N-Tg(MMTV-PyVT)634Mul/J, gift from Kristian Pietras) (Guy et al., 1992) were used to evaluate PAK4 expression in mammary tumors and adjacent mammary tissue and to study the intratumoral effects of PF-03758309.<br>Female 6 to 8 weeks old BALB/c mice (CAnN.Cg-Foxn1nu/Crl, Charles River) were used in the xenograft model. BALB/c mice acclimatized for 1 week before being randomly assigned per experimental condition.
Wild animals	N/A
Field-collected samples	N/A
Ethics oversight	Ethical approval was provided from the Stockholms Södra and Linköpings djurförsöksetiska nämnder.

Note that full information on the approval of the study protocol must also be provided in the manuscript.
